# Surveying the Down syndrome mouse model resource identifies critical regions responsible for chronic otitis media

**DOI:** 10.1007/s00335-013-9475-x

**Published:** 2013-09-26

**Authors:** Mahmood F. Bhutta, Michael T. Cheeseman, Yann Herault, Yuejin E. Yu, Steve D. M. Brown

**Affiliations:** 1 Nuffield Department of Surgical Sciences, John Radcliffe Hospital, University of Oxford, Room 6607 Level 6, Headley Way, Oxford, OX3 9DU UK; 2 MRC Harwell, Harwell Science and Innovation Campus, Oxfordshire, OX11 0RD UK; 3 Department of Otolaryngology Head and Neck Surgery, John Radcliffe Hospital, Level LG1 West Wing, Headley Way, Oxford, OX3 9DU UK; 4 Institut de Génétique, Biologie Moléculaire et Cellulaire et Institut, Clinique de la Souris, 1 rue Laurent Fries, Parc d’Innovation, BP 10142, 67404 Illkirch-Graffenstaden Cedex, France; 5 Department of Cancer Genetics, CGP L2-320, Roswell Park Cancer Institute, Elm & Carlton Streets, Buffalo, NY 14263 USA

## Abstract

Chronic otitis media (OM) is common in Down syndrome (DS), but underlying aetiology is unclear. We analysed the entire available mouse resource of partial trisomy models of DS looking for histological evidence of chronic middle-ear inflammation. We found a highly penetrant OM in the Dp(16)1Yey mouse, which carries a complete trisomy of MMU16. No OM was found in the Dp(17)1Yey mouse or the Dp(10)1Yey mouse, suggesting disease loci are located only on MMU16. The Ts1Cje, Ts1RhR, Ts2Yah, and Ts65Dn trisomies and the transchomosomic Tc1 mouse did not develop OM. On the basis of these findings, we propose a two-locus model for chronic middle-ear inflammation in DS, based upon epistasis of the regions of HSA21 not in trisomy in the Tc1 mouse. We also conclude that environmental factors likely play an important role in disease onset.

## Introduction

Down syndrome (DS) is the most common human chromosomal abnormality, occurring in 1/750 live births, and is due to partial or total trisomy HSA21. Variable clinical features include skull and midface malformation, cardiac anomalies, hypotonia, delayed growth, developmental disorders, thyroid disease, obesity, and also chronic otitis media (OM). Most children with DS develop OM with effusion (OME), which is often chronic (COME) and leads to a hearing loss that can compound learning difficulties (Libb et al. 1985; Marcell and Cohen 1992). A longitudinal study of 79 children with DS reported that OME was diagnosed in 93 % at age one, falling to 68 % by age 5 (Barr et al. 2011). Similar findings have been reported in other series (Brooks et al. 1972; Balkany et al. 1979; Maurizi et al. 1985; Dahle and McCollister 1986; Hassmann et al. 1998; Kattan et al. 2000; Mitchell et al. 2003; Park et al. 2011). COME may persist, and at least 10–20 % of adults with DS have a conductive hearing loss (Evenhuis et al. 1992; Van Buggenhout et al. 1999). Middle-ear inflammation may also be subclinical (Roizen et al. 1994). Several authorities advise routine and regular audiological screening of children with DS (American Academy of Pediatrics 2001; Downs Syndrome Medical Interest Group 2004).

Standard treatments for COME are not as effective in children with DS. Grommets (ventilation tubes) in this population may not significantly improve hearing, have little if any effect on disease recurrence, and are not infrequently complicated by infection (Selikowitz 1993; Iino et al. 1999; Shott et al. 2001). Some advocate hearing aids as the preferred treatment for COME in DS (National Institute for Health and Clinical Excellence 2008). An understanding of the pathogenic mechanisms leading to chronic middle-ear inflammation in DS offers potential for targeted and more effective therapy of COME in this population. It may also provide insights into mechanisms of inflammation in nonsyndromic COME, which is also highly heritable (Casselbrant et al. 1999). Specifically, trisomy in DS could mimic hypermorphic polymorphisms underlying nonsyndromic disease.

The aetiology of chronic middle-ear inflammation in DS is not understood. Children with DS are more prone to infection as a result of defective mucosal immunology (Kusters et al. 2009). This could explain the onset of OM, but the persistent nonsuppurative inflammation seen in COME is not thought to reflect persistent bacterial infection. Several authorities suggest that an anatomical anomaly of the Eustachian tube in DS contributes to nonresolving disease (Bluestone et al. 2005), but the only published study on Eustachian tube patency in DS did not demonstrate the tube to be obstructed (White et al. 1984).

DS is presumed to arise as a consequence of either an extra copy of protein-coding sequences that are dosage-sensitive or an extra copy of non-protein-coding sequences that are regulatory or otherwise functional (Antonarakis et al. 2004; Gardiner and Costa 2006; Megarbane et al. 2009; Tan et al. 2009; Wiseman et al. 2009). HSA21 is the smallest human autosome, with only around 300 genes, and so perhaps only a handful of genes play a role in the phenotypic manifestations of DS. Individuals with partial trisomy of HSA21 offer serendipitous clues to identify critical regions for DS phenotypes. Initial analyses of such individuals suggested that all or most phenotypic manifestations of DS were due to a short critical region (McCormick et al. 1989; Rahmani et al. 1989), but more recent analysis refutes this and suggests that loci determining the DS phenotype are scattered across HSA21 (Lyle et al. 2004; Korbel et al. 2009). This analysis had not been undertaken specifically for the OM phenotype, but given the rarity of partial trisomy HSA21 and the lack of a large database of genotype-phenotype correlation for aneuploidies, such analysis is difficult at present.

Readouts of the transcriptome of HSA21 have also been used to define critical genes. Data from human (Ait Yahya-Graison et al. 2007) and mouse (Lyle et al. 2004) show that only around a third of the genes on HSA21 are transcribed at more than the theoretical 1.5-fold increase, suggesting that the majority of trisomic loci are compensated for by negative feedback that modulates transcriptional activity or mRNA stability. Although this does appear to narrow the possibilities, the method is not entirely reliable. Data of the transcriptome from mouse and human differ, as do data from different tissue types (Lyle et al. 2004). In addition, transcription is but one component of gene expression (Schwanhausser et al. 2011), so small changes in transcription could have a large phenotypic effect at some loci, and large changes in transcription could have little or no phenotypic effect at other loci.

**Fig. 1:**
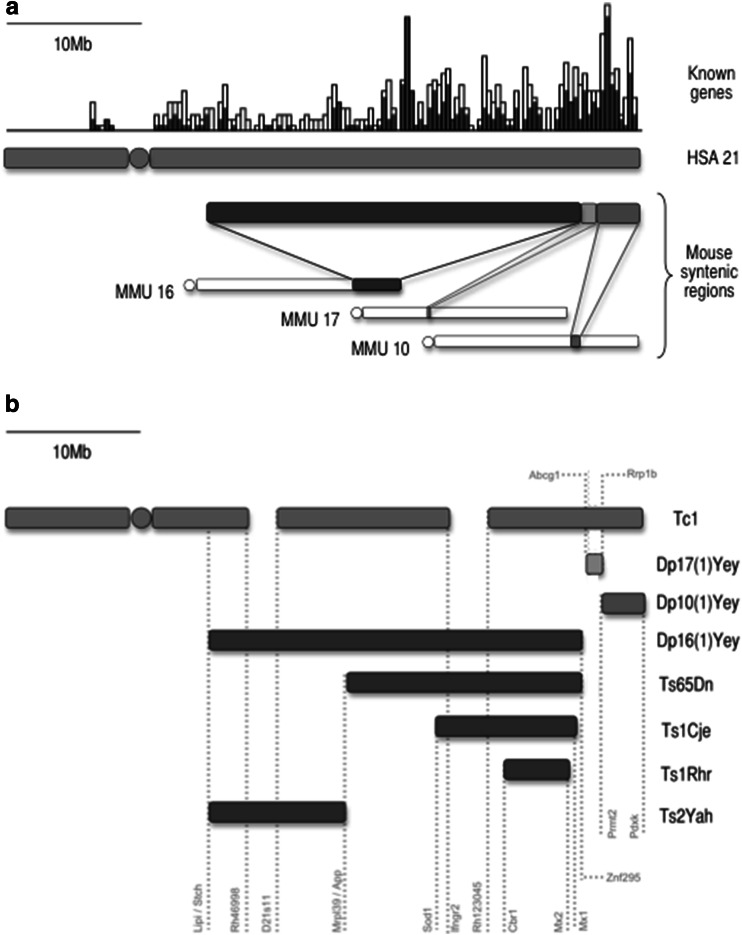
Map of **a** murine synteny to human chromosome 21 and of **b** trisomic regions in mouse models of DS used in this study (showing genes at chromosome breakpoints). The syntenic regions to HSA21 are on MMU16 (76–98 Mb), MMU17 (31–32 Mb), and MMU10 (78–76 Mb). The Tc1 mouse carries an incomplete HSA21 in addition to the normal mouse genome, whereas all other models are trisomic for murine DNA. Known genes are derived from the Ensembl web browser

There has been increasing interest in using mouse models to unravel pathobiology in DS. The syntenic regions to HSA21 are scattered across the mouse genome on MMU16, MMU17, and MMU10 (see Fig. 1a), which makes genetic modelling difficult (Gardiner et al. 2003). However, there is a growing library of mouse models of DS. The Ts65Dn mouse (Davisson et al. 1993), Ts1Cje mouse (Sago et al. 1998), Ts2Yah mouse (Herault et al. 2009), Ts1Rhr mouse (Olson et al. 2004), Dp(16)1Yey mouse (Li et al. 2007), Dp(10)1Yey mouse (Yu et al. 2010), and Dp(17)1Yey mouse (Yu et al. 2010) each carry a partial trisomy for the homologous genes of HSA21. Together these models cover almost all regions triplicated in DS (Fig. 1b). In addition, the Tc1 mouse is a transchromosomic model, carrying most of HSA21 in addition to the full complement of mouse chromosomes (O’Doherty et al. 2005).

This mouse library has been surveyed to identify loci responsible for some of the phenotypic manifestations of DS, but it has never been systematically interrogated for the presence of OM. The Ts65Dn mouse, which carries a 15.6-Mb segmental trisomy of MMU16 (Duchon et al. 2011; Reinholdt et al. 2011), has been reported to develop spontaneous chronic OM (Han et al. 2009), and recently the Tc1 mouse has been reported to not develop chronic OM (Kuhn et al. 2012). There are no reports of the presence or absence of OM in the other murine models of DS. In this study we systematically screened the murine resource of DS models to identify the histological presence of chronic OM, with the aim of narrowing and defining a critical region or set of loci underlying this phenotype.

## Materials and methods

### Mouse strains

We analysed cadaveric specimens of almost the entire reported library of DS mouse models. Specimens of the Ts65Dn mouse, the Ts1Cje mouse, the Ts2Yah mouse, and the Ts1Rhr mouse were obtained from the Institut Clinique de la Souris, Strasbourg. Specimens of the Dp(16)1Yey mouse, the Dp(10)1Yey mouse, and the Dp(17)1Yey mouse were obtained from Roswell Park, NY, USA. Specimens of the Tc1 mouse and additional specimens of the Ts65Dn mouse were obtained from University College London. The number and age of mice supplied depended upon local availability. All mice were supplied with wild-type (WT) littermate controls, and all were genotyped at source. The microbial status and housing conditions of colony mice varied between institutions, as detailed in Table 1.

**Table 1:** Microbial status of mouse strains

Source	Mouse lines	FELASA listed microbial agents	Housing conditions
Institut Clinique de la Souris	Ts65Dn, Ts1Cje, Ts2Yah, Ts1Rhr	Norovirus, *Helicobacter* spp., *Pseudomonas aeruginosa*, *Entamoeba muris*	Individually ventilated cages with HEPA-filtered air
Roswell Park	Dp(10)1Yey, Dp(16)1Yey, Dp(17)1Yey	Norovirus, *Helicobacter* spp.	Individually ventilated cages with HEPA-filtered air
University College London	Tc1, Ts65Dn	*Helicobacter hepaticus*, *Tritrichomonas*	Open cages

*FELASA* Federation for Laboratory Animal Science Associations

### Histology

Mouse heads were fixed in 10 % neutral buffered formalin, decalcified, and embedded in paraffin wax. Three-micron sections were stained with haematoxylin and eosin and assessed for histological evidence of inflammation of the middle ear (Cheeseman et al. 2011).

### Statistics

We used Fisher’s exact test to compare prevalence of OM in mutant versus WT littermates.

## Results

We examined serial sections of mouse heads from eight mouse lines [Ts65Dn, Ts1Cje, Ts2Yah, Ts1Rhr, Dp(16)1Yey, Dp(10)1Yey, Dp(17)1Yey, and Tc1] for histological evidence of inflammation of the middle ear. A total of 532 ears were analysed and the results are documented in Table 2. For one mouse line, Ts65Dn, mice were gathered from two sources.

**Table 2:** Mouse strains used in this study, their source, and presence or absence of OM on histology (denominator is for number of ears)

Mouse line	Background strain	Trisomic segment	Source	Age range (months)	OM in mutant	OM in WT controls	*p* value
Dp(17)1Yey	B6;129	MMU17 *Abcg1-Rrp1b*	Roswell Park	2–3	0/28	1/32	1
Dp(10)1Yey	B6;129	MMU10 *Prmt2-Pdxk*	Roswell Park	2–4	0/28	0/28	1
Dp(16)1Yey	B6;129	MMU16 *Lipi* *-Znf295*	Roswell Park	2–15	19/26	7/24	0.004
Ts1Cje	B6	MMU16 *Sod1-Znf295*	ICS	2	2/28	0/28	0.491
Ts1RhR	B6	MMU16 *Cbr1-Mx2*	ICS	2	0/36	0/36	1
Ts2Yah	B6	MMU16 *Stch -App*	ICS	2	0/34	0/34	1
Ts65Dn	C3H;B6	MMU16 *Mrpl39-Znf295* & MMU17 *Synj2-D17Mit58*	ICS	2	2/40^a^	0/40	0.494
Ts65Dn	C3H;B6	MMU16 *Mrpl39-Znf295* & MMU17 *Synj2-D17Mit58*	UCL	11–16	1/12^b^	0/22	0.353
Tc1	129;B6	HSA21 except *Rh46998-D21s11* & *Ifngr2-Rh120345*	UCL	2–3	0/30	0/26	1

The *p* values relate to Fisher’s exact test for the difference in OM prevalence in mutants versus wild-type (WT) littermates

*UCL* University College London, *ICS* Institut Clinique de la Souris

^a^Two ears in this cohort of Ts65Dn mice showed mild oedema of the middle-ear mucoperiosteum, but without leucocyte infiltration

^b^One ear showed a small middle-ear exudate with micropolyp formation

**Fig. 2:**
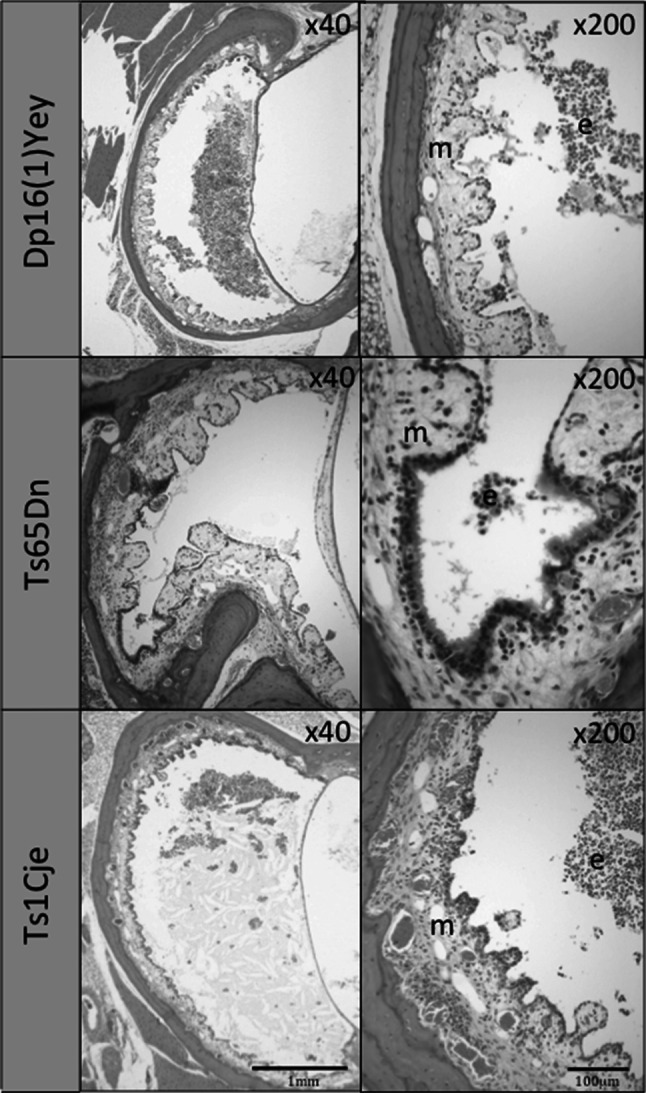
Histological section through the middle ear of a Dp(16)1Yey mouse, a Ts65Dn mouse, and a Ts1Cje mouse affected by chronic OM (H&E stain). There is polypoid hyperplasia of the mucoperiosteum (m) with an effusion (e) infiltrated by neutrophils and macrophages. This was present in 19/26 of Dp(16)1Yey mice examined, 3/52 of Ts65Dn mice, and 1/14 Ts1Cje mice

Only the Dp(16)1Yey mouse line reliably developed statistically significant chronic OM (19 ears in 10 of 13 mice). Histology revealed thickened polypoid mucoperiosteum with neutrophil and macrophage infiltration, and an effusion of variable cellularity (Fig. 2). In this line, disease was consistently present from the age of 2 months. Some of the older (>7 months old) WT littermate controls for this line also developed chronic OM (7 ears in 4 mice), but OM was not present in any of the younger WT littermates.

OM in other models was not present or it was rarely present. Given the reported OM in T65Dn mice, we obtained animals from two different sources. We found no evidence of OM in either colony. A unilateral small volume of exudate and inflammatory polyp formation were noted in one of the Ts65Dn mice from University College London (Fig. 2). There was also mild unilateral oedema in the middle-ear mucosa of two Ts65Dn mice from the Institut Clinique de la Souris, but without a leucocyte infiltrate (not shown). There was bilateral OM in one Ts1Cje mouse, with infiltration by macrophages and neutrophils (Fig. 2).

## Discussion

### Chromosomal localisation of OM

In this study we screened the DS mouse library to identify a critical region for genetic susceptibility to chronic OM. The Dp(16)1Yey mouse, which is trisomic for the entire region of MMU16 syntenic to HSA21, was found to reliably develop chronic OM. Here, OM was early onset (by 2 months) and with penetrance of 0.73 (19/26 ears). A few of the WT littermate controls of the Dp(16)1Yey mice also developed OM, but only when they were older than 7 months. Chronic OM is common in older laboratory mice (Haines et al. 2001), although the reasons are unclear, and so the pathology in the older WT mice is not unexpected.

Of note, there was no evidence of OM in the Dp(17)1Yey and Dp(10)1Yey models, suggesting that only genes found on MMU16 are responsible for the OM phenotype. We also found no evidence of OM in the Tc1 mouse, in agreement with recently reported results (Kuhn et al. 2012). The Tc1 model carries many of the genes found in trisomy in DS (in the form of mouse and human chromosomes), but it has been reported to lack many elements of the DS phenotype. It has been surmised that this may reflect species differences in genetic regulatory elements, or it may be the result of genetic mosaicism (Patterson 2009). More recent work also suggests previously unrecognised gene rearrangements in the Tc1 mouse (E. Fisher, personal communication).

Our findings strongly implicate a region of MMU16 to underlie the chronic OM phenotype. Empirically, this region could be narrowed further by analysis of the Ts65Dn, Ts1Cje, Ts1Rhr, and Ts2Yah mice, each of which carry shorter segments trisomic for MMU16 (Fig. 1).

### Localisation of OM within MMU16

We found no OM in Ts1Rhr and Ts2Yah mice, but one Ts1Cje mouse developed severe bilateral OM. We observed definitive OM in only one ear of the 52 Ts65Dn ears screened, although there was mild mucosal oedema in two other Ts65Dn mouse ears (which may be indicative of early or mild inflammation). The absence of OM in these four mouse lines, coupled with the highly penetrant OM observed in the Dp(16)1Yey mouse, suggests a genetic model for OM in DS whereby more than one locus is required. Given the absence of OM in Tc1, this would suggest that the loci are present in the two chromosome 16 regions not found in Tc1, i.e., *Rh46998-D21s11* and *Ifngr2-Rh123045* (Fig. 1b). Known genes in these two regions and their function are detailed in Table 3. The first region contains 4 genes, of which only *Cxadr* is known to be involved in immune signalling. The second region contains 17 genes, of which two, *Ifngr2* and *Runx1*, are known to be involved in immune signalling. *Cxadr* and *Ifngr2* are usually invoked in response to viral rather than bacterial infection. Whether these or other loci in these regions are responsible for persistent middle-ear inflammation we can only surmise.

**Table 3:** Genes located in the regions *Rh46998-D21s11* (region A) and *Ifngr2-Rh123045* (region B)

Gene	Relative transcript level	Function	Candidate
Region A			
* CXADR*	?	Coxsackie and adenovirus receptor, maintains tight junctions	✠
* C21ORF*	?	Unknown	
* CHODL*	?	Endocytosis of glycoproteins and sugar-bearing pathogens	
* TMPRSS15*	?	Pancreatic enzyme	
Region B			
* IFNGR2*	1.37	Receptor subunit for interferon γ	✠
* TMEM50B*	1.43	Brain development	
* DNAJC28*	–	May act in protein folding or as a chaperone	
* GART*	1.46	Purine biosynthesis	
* SON*	1.77	mRNA splicing cofactor	
* DONSON*	1.35	Unknown	
* CRYZL1*	1.40	Unknown	
* ITSN1*	1.42	Coordinates endocytic membrane traffic	
* ATP5O*	1.40	ATP synthesis	
* MRPS6*	1.28	Mitochondrial protein synthesis	
* SLC5A3*	0.91	Prevents intracellular accumulation of myo-inositol	
* KCNE2*	1.32	Component of voltage-gated potassium channels	
* FAM165B*	1.39	Unknown	
* KCNE1*	–	Component of voltage-gated potassium channels	
* RCAN1*	0.79	Inhibits calcineurin-dependent signaling pathways	
* CLIC6*	–	Probable chloride ion channel	
* RUNX1*	1.31	Functions in haematopoeisis, interacts with TGF-β	✠

Regions A and B are trisomic in the Dp(16)1Yey mouse (develops OM), but not in the Tc1 mouse (no OM noted). Relative transcript levels for region B refer to those in adult lung of Ts65Dn mice (Lyle et al. 2004); region A is not trisomic in Ts65Dn mice. In the proposed multigenic model, genes from each of these regions interact to produce susceptibility to chronic OM. Highlighted “candidates” are those genes known to be involved in immune signaling

In considering this multigenic model, it is important to examine the differing results reported in this study and those of Han et al. (2009) regarding the incidence of OM observed in the Ts65Dn mouse. Han et al. reported a highly penetrant chronic OM in the Ts65Dn model (in 11/15 mice), with a variable cellular infiltrate and effusion, accompanied by audiological evidence of disease onset by the age of 2 months. The variable penetrance of OM in the Ts65Dn mouse observed in this and the Han study suggests that development of chronic OM in DS is not determined solely by a critical genetic region(s) in trisomy, but also by additional important modifiers of phenotype, possibly genetic, environmental, or both.

Background sequence variation could affect phenotype penetrance and expressivity. The Ts65Dn mice in our study were maintained on a C3;B6 background through breeding with local stock. The Ts65Dn mice reported by Han et al. were similarly maintained by local breeding on a C3;B6 background. However, even small genomic variations can play a pivotal role in phenotype penetrance in DS (Antonarakis et al. 2004) and thus interlaboratory variation might affect the OM phenotype. For example, allelic variation in *CRELD1* on HSA3 modifies the risk of cardiac malformation in children (Maslen et al. 2006). In the mouse, breeding the Ts1RhR mouse model onto a mixed B6;C3H/129 background, as opposed to pure B6, leads to loss of the craniofacial phenotype (Keane et al. 2011). However, it is noteworthy that we obtained T65Dn mice from two separate sources, and in neither case did we observe OM.

An alternative, and perhaps more likely, explanation is that an environmental antigen, such as a bacterial pathogen, can trigger the onset of OM in Ts65Dn mice. Variation in housing conditions could lead to differences in environmental exposure and so explain variable phenotype penetrance. Han et al. (2009) reported coagulase-negative *Staphylococci*, *Burkholderia cepacia*, and *Bordetella avium* in middle-ear effusions of their Ts65Dn mice, but also *Klebsiella oxytoca*, *Streptococcus viridans*, and *B. avium* in wild-type controls. We did not culture bacteria from effusions in this study (only fixed tissues were used), but because a number of microbes have been isolated at the source of these animals (Table 2), it is possible that one or more of these microbes, or other unidentified microbes, play a pivotal role in disease onset.

In summary, our data suggest a multigenic model for development of OM in DS mice, whereby loci from two chromosome 16 regions contribute to the onset of the disease. Consistent with our proposed model, we failed to observe definitive OM in the Ts65Dn mouse, which would carry only one of the required chromosome 16 regions. However, it may be that environmental insults or other genetic variation elsewhere in the genome may be sufficient along with the trisomic region present in Ts65Dn mouse to elicit OM. This would potentially account for the differences in incidence of OM observed between the work of Han et al. and the work reported here.
